# Surgical Applied Anatomy

**Published:** 1884-09

**Authors:** 


**Surgical Applied Anatomy.** By FRED. TREVES, F. R. C. S., Senior Demonstrator of Anatomy at the London Hospital. Illustrated with 61 engravings. H. C. Lea's Son & Co., Philadelphia.

